# Response to “Comment on ‘Independent corroboration of monitor unit calculations performed by a 3D computerized planning system’” [J. Appl. Clin. Med. Phys. 2, 102 (2001)]

**DOI:** 10.1120/jacmp.v2i2.2619

**Published:** 2001-03-01

**Authors:** Konrad W. Leszczynski, Peter B. Dunscombe

**Affiliations:** ^1^ Department of Medical Physics Northeastern Ontario Regional Cancer Center 41 Ramsey Lake Rd. Sudbury P3E 5J1 Canada; ^2^ Department of Radiology, University of Ottawa Department of Physics Laurentian University

## Abstract

PACS number(s): 87.53.–j, 87.52.–g

To the Editor,

We wish to thank the reader for his interest in our study. While his comments on the different factors that affect the accuracy of manual dose calculations are generally valid, we do not see that they have been in any way ignored in our work. It does appear to us, however, that the reader has missed some of the important points made in our article, in particular the distinction between the accuracy and the precision of the manual dose calculation method. We argue that even though a practicable manual calculation method may not match the accuracy of a 3D computerized planning system, it can still be useful as a quality assurance tool as long as its precision is adequately high and the systematic inaccuracies are well understood. In our article we discuss the observed shortcomings in the accuracy of our manual checks and explain them in terms of the underlying simplifications that were applied in our method. In doing so we identified some of the factors mentioned by the reader (e.g., off‐axis ratio, beam hardening) as the sources of inaccuracies, while other factors, although potentially significant under some circumstances, clearly did not play a major role in the cases covered by our analysis. Another important fact to remember while considering the reader's arguments is that the quoted 2% to 4% dose errors occur for single beam calculations and for dose points placed somewhat extremely, e.g., at shallow depths and far away from the central axis. The results quoted in our article refer to comparisons of total doses, received by dose reference points from multiple beams. Thus a 2% error from one beam would not necessarily result in a significant error in the total dose. Furthermore, as we indicated, the reference points were defined in accordance with ICRU 50 recommendations. These recommendations specifically call for placement of the reference point in a region where the dose can be accurately determined–on or near the central axis.

Despite the difference in views between the reader and the authors as to the necessary degree of complexity in “manual” MU calculations we all agree that it is a very important component of quality assurance in radiation therapy. Furthermore, our study shows that even a simplified manual calculation method provides satisfactory precision for detection of significant dosimetric errors in treatment planning.

